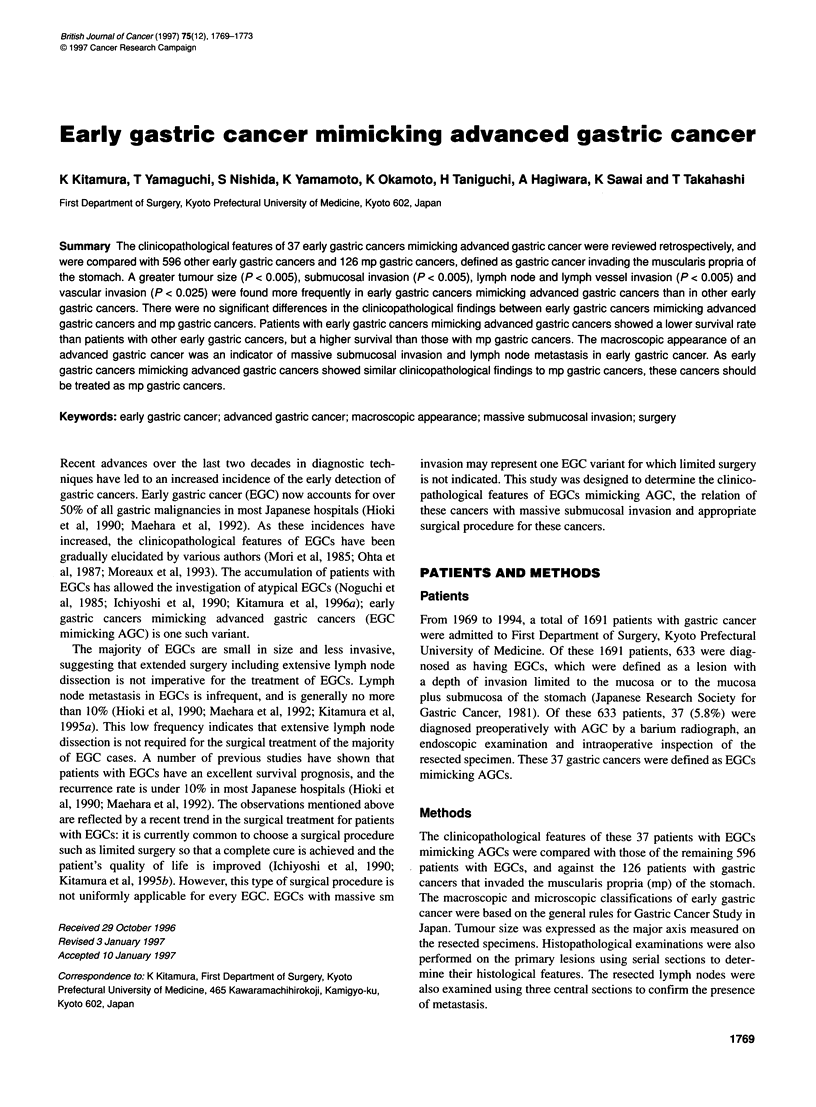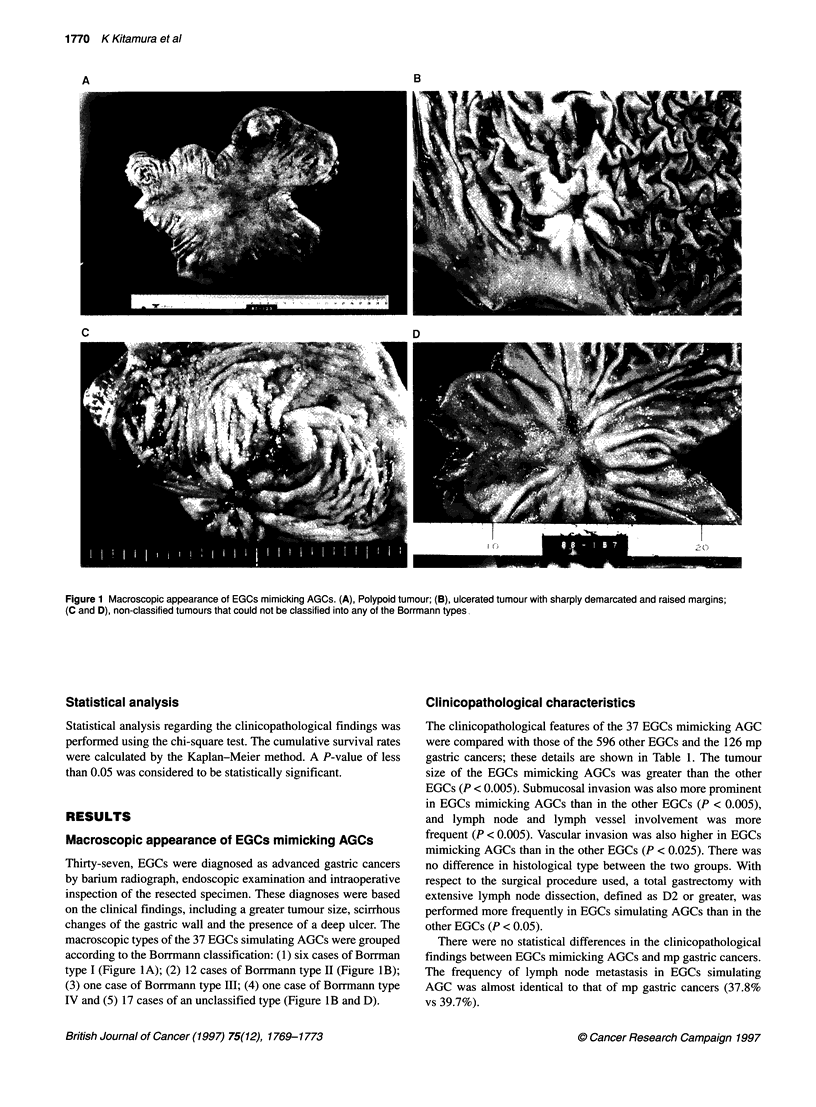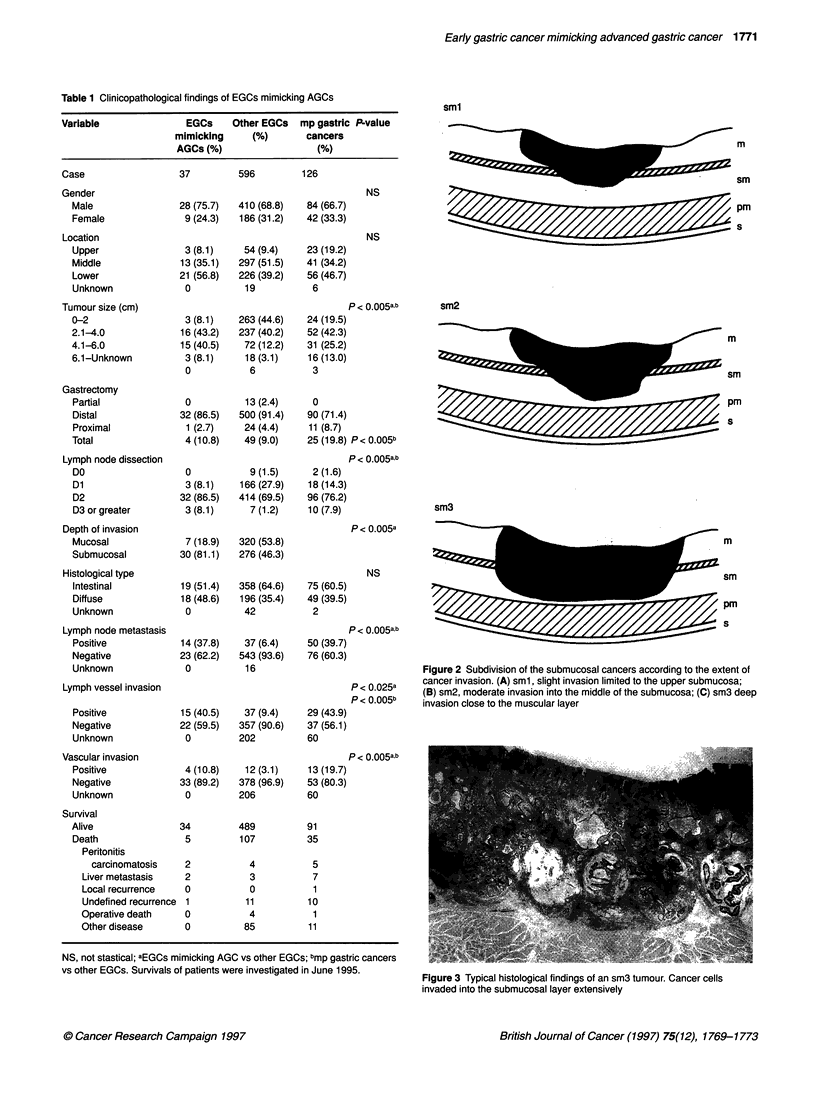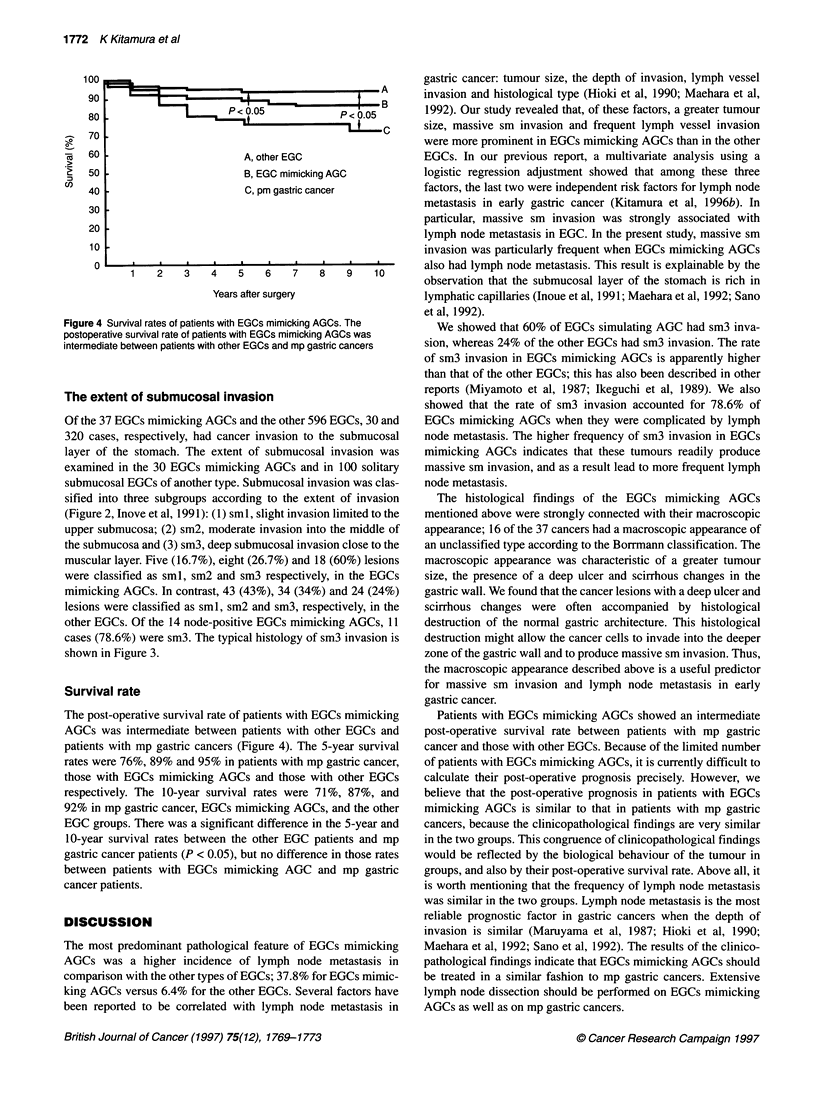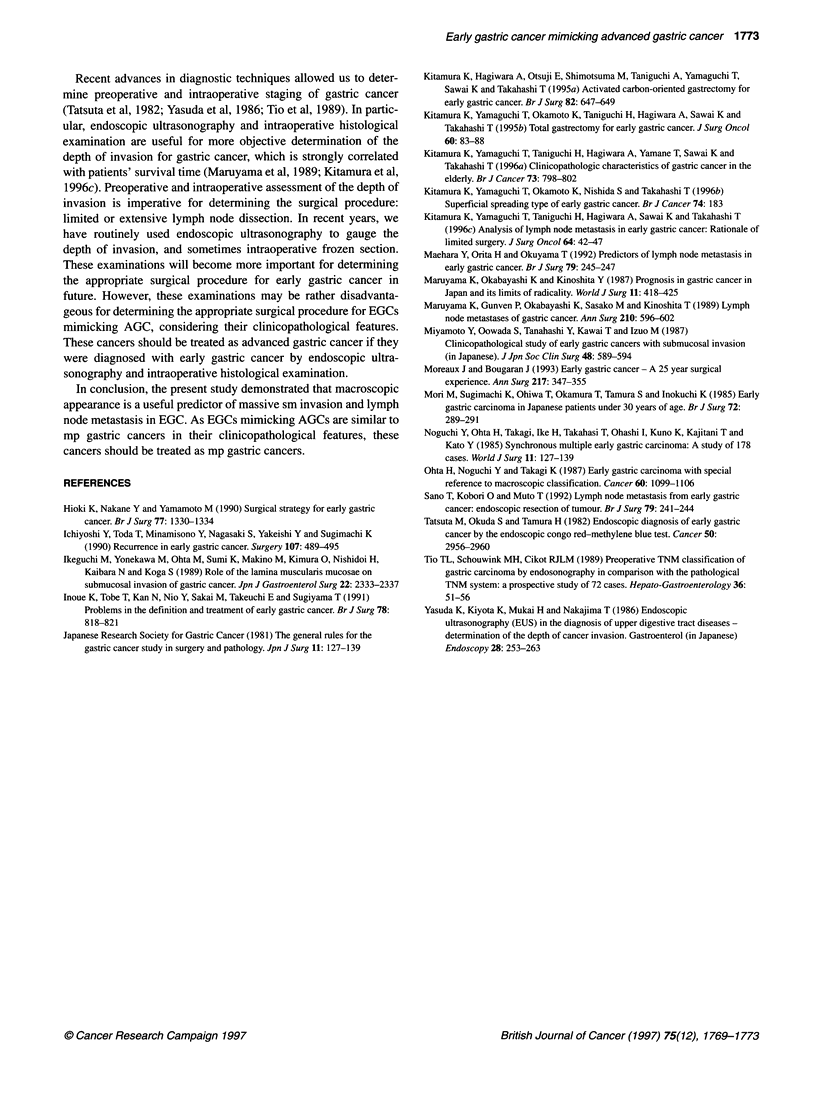# Early gastric cancer mimicking advanced gastric cancer.

**DOI:** 10.1038/bjc.1997.301

**Published:** 1997

**Authors:** K. Kitamura, T. Yamaguchi, S. Nishida, K. Yamamoto, K. Okamoto, H. Taniguchi, A. Hagiwara, K. Sawai, T. Takahashi

**Affiliations:** First Department of Surgery, Kyoto Prefectural University of Medicine, Kamigyo-ku, Japan.

## Abstract

**Images:**


British Journal of Cancer (1997) 75(12), 1769–1773
© 1997 Cancer Research Campaign

### Early gastric cancer mimicking advanced gastric cancer

**K Kitamura, T Yamaguchi, S Nishida, K Yamamoto, K Okamoto, H Taniguchi, A Hagiwara, K Sawai and T Takahashi**

First Department of Surgery, Kyoto Prefectural University of Medicine, Kyoto 602, Japan

**Summary** The clinicopathological features of 37 early gastric cancers mimicking advanced gastric cancer were reviewed retrospectively, and were compared with 596 other early gastric cancers and 126 mp gastric cancers, defined as gastric cancer invading the muscularis propria of the stomach. A greater tumour size ($P < 0.005$), submucosal invasion ($P < 0.005$), lymph node and lymph vessel invasion ($P < 0.005$) and vascular invasion ($P < 0.025$) were found more frequently in early gastric cancers mimicking advanced gastric cancers than in other early gastric cancers. There were no significant differences in the clinicopathological findings between early gastric cancers mimicking advanced gastric cancers and mp gastric cancers. Patients with early gastric cancers mimicking advanced gastric cancers showed a lower survival rate than patients with other early gastric cancers, but a higher survival than those with mp gastric cancers. The macroscopic appearance of an advanced gastric cancer was an indicator of massive submucosal invasion and lymph node metastasis in early gastric cancer. As early gastric cancers mimicking advanced gastric cancers showed similar clinicopathological findings to mp gastric cancers, these cancers should be treated as mp gastric cancers.

**Keywords:** early gastric cancer; advanced gastric cancer; macroscopic appearance; massive submucosal invasion; surgery

Recent advances over the last two decades in diagnostic techniques have led to an increased incidence of the early detection of gastric cancers. Early gastric cancer (EGC) now accounts for over 50% of all gastric malignancies in most Japanese hospitals (Hioki et al, 1990; Maehara et al, 1992). As these incidences have increased, the clinicopathological features of EGCs have been gradually elucidated by various authors (Mori et al, 1985; Ohta et al, 1987; Moreaux et al, 1993). The accumulation of patients with EGCs has allowed the investigation of atypical EGCs (Noguchi et al, 1985; Ichiyoshi et al, 1990; Kitamura et al, 1996*a*); early gastric cancers mimicking advanced gastric cancers (EGC mimicking AGC) is one such variant.

The majority of EGCs are small in size and less invasive, suggesting that extended surgery including extensive lymph node dissection is not imperative for the treatment of EGCs. Lymph node metastasis in EGCs is infrequent, and is generally no more than 10% (Hioki et al, 1990; Maehara et al, 1992; Kitamura et al, 1995*a*). This low frequency indicates that extensive lymph node dissection is not required for the surgical treatment of the majority of EGC cases. A number of previous studies have shown that patients with EGCs have an excellent survival prognosis, and the recurrence rate is under 10% in most Japanese hospitals (Hioki et al, 1990; Maehara et al, 1992). The observations mentioned above are reflected by a recent trend in the surgical treatment for patients with EGCs: it is currently common to choose a surgical procedure such as limited surgery so that a complete cure is achieved and the patient's quality of life is improved (Ichiyoshi et al, 1990; Kitamura et al, 1995*b*). However, this type of surgical procedure is not uniformly applicable for every EGC. EGCs with massive sm invasion may represent one EGC variant for which limited surgery is not indicated. This study was designed to determine the clinicopathological features of EGCs mimicking AGC, the relation of these cancers with massive submucosal invasion and appropriate surgical procedure for these cancers.

*Received 29 October 1996*
*Revised 3 January 1997*
*Accepted 10 January 1997*

*Correspondence to:* K Kitamura, First Department of Surgery, Kyoto Prefectural University of Medicine, 465 Kawaramachihirokoji, Kamigyo-ku, Kyoto 602, Japan

### PATIENTS AND METHODS

### Patients

From 1969 to 1994, a total of 1691 patients with gastric cancer were admitted to First Department of Surgery, Kyoto Prefectural University of Medicine. Of these 1691 patients, 633 were diagnosed as having EGCs, which were defined as a lesion with a depth of invasion limited to the mucosa or to the mucosa plus submucosa of the stomach (Japanese Research Society for Gastric Cancer, 1981). Of these 633 patients, 37 (5.8%) were diagnosed preoperatively with AGC by a barium radiograph, an endoscopic examination and intraoperative inspection of the resected specimen. These 37 gastric cancers were defined as EGCs mimicking AGCs.

### Methods

The clinicopathological features of these 37 patients with EGCs mimicking AGCs were compared with those of the remaining 596 patients with EGCs, and against the 126 patients with gastric cancers that invaded the muscularis propria (mp) of the stomach. The macroscopic and microscopic classifications of early gastric cancer were based on the general rules for Gastric Cancer Study in Japan. Tumour size was expressed as the major axis measured on the resected specimens. Histopathological examinations were also performed on the primary lesions using serial sections to determine their histological features. The resected lymph nodes were also examined using three central sections to confirm the presence of metastasis.

**Figure 1** Macroscopic appearance of EGCs mimicking AGCs. (**A**), Polypoid tumour; (**B**), ulcerated tumour with sharply demarcated and raised margins; (**C** and **D**), non-classified tumours that could not be classified into any of the Borrmann types.

### Statistical analysis

Statistical analysis regarding the clinicopathological findings was performed using the chi-square test. The cumulative survival rates were calculated by the Kaplan–Meier method. A *P*-value of less than 0.05 was considered to be statistically significant.

### RESULTS

### Macroscopic appearance of EGCs mimicking AGCs

Thirty-seven, EGCs were diagnosed as advanced gastric cancers by barium radiograph, endoscopic examination and intraoperative inspection of the resected specimen. These diagnoses were based on the clinical findings, including a greater tumour size, scirrhous changes of the gastric wall and the presence of a deep ulcer. The macroscopic types of the 37 EGCs simulating AGCs were grouped according to the Borrmann classification: (1) six cases of Borrman type I (Figure 1A); (2) 12 cases of Borrmann type II (Figure 1B); (3) one case of Borrmann type III; (4) one case of Borrmann type IV and (5) 17 cases of an unclassified type (Figure 1B and D).

### Clinicopathological characteristics

The clinicopathological features of the 37 EGCs mimicking AGC were compared with those of the 596 other EGCs and the 126 mp gastric cancers; these details are shown in Table 1. The tumour size of the EGCs mimicking AGCs was greater than the other EGCs ($P < 0.005$). Submucosal invasion was also more prominent in EGCs mimicking AGCs than in the other EGCs ($P < 0.005$), and lymph node and lymph vessel involvement was more frequent ($P < 0.005$). Vascular invasion was also higher in EGCs mimicking AGCs than in the other EGCs ($P < 0.025$). There was no difference in histological type between the two groups. With respect to the surgical procedure used, a total gastrectomy with extensive lymph node dissection, defined as D2 or greater, was performed more frequently in EGCs simulating AGCs than in the other EGCs ($P < 0.05$).

There were no statistical differences in the clinicopathological findings between EGCs mimicking AGCs and mp gastric cancers. The frequency of lymph node metastasis in EGCs simulating AGC was almost identical to that of mp gastric cancers (37.8% vs 39.7%).

© Cancer Research Campaign 1997

**Table 1** Clinicopathological findings of EGCs mimicking AGCs

| Variable | EGCs mimicking AGCs (%) | Other EGCs (%) | mp gastric cancers (%) | P-value |
|---|---|---|---|---|
| Case | 37 | 596 | 126 | |
| Gender | | | | NS |
| Male | 28 (75.7) | 410 (68.8) | 84 (66.7) | |
| Female | 9 (24.3) | 186 (31.2) | 42 (33.3) | |
| Location | | | | NS |
| Upper | 3 (8.1) | 54 (9.4) | 23 (19.2) | |
| Middle | 13 (35.1) | 297 (51.5) | 41 (34.2) | |
| Lower | 21 (56.8) | 226 (39.2) | 56 (46.7) | |
| Unknown | 0 | 19 | 6 | |
| Tumour size (cm) | | | | P < 0.005[a,b] |
| 0–2 | 3 (8.1) | 263 (44.6) | 24 (19.5) | |
| 2.1–4.0 | 16 (43.2) | 237 (40.2) | 52 (42.3) | |
| 4.1–6.0 | 15 (40.5) | 72 (12.2) | 31 (25.2) | |
| 6.1–Unknown | 3 (8.1) | 18 (3.1) | 16 (13.0) | |
| | 0 | 6 | 3 | |
| Gastrectomy | | | | |
| Partial | 0 | 13 (2.4) | 0 | |
| Distal | 32 (86.5) | 500 (91.4) | 90 (71.4) | |
| Proximal | 1 (2.7) | 24 (4.4) | 11 (8.7) | |
| Total | 4 (10.8) | 49 (9.0) | 25 (19.8) | P < 0.005[b] |
| Lymph node dissection | | | | P < 0.005[a,b] |
| D0 | 0 | 9 (1.5) | 2 (1.6) | |
| D1 | 3 (8.1) | 166 (27.9) | 18 (14.3) | |
| D2 | 32 (86.5) | 414 (69.5) | 96 (76.2) | |
| D3 or greater | 3 (8.1) | 7 (1.2) | 10 (7.9) | |
| Depth of invasion | | | | P < 0.005[a] |
| Mucosal | 7 (18.9) | 320 (53.8) | | |
| Submucosal | 30 (81.1) | 276 (46.3) | | |
| Histological type | | | | NS |
| Intestinal | 19 (51.4) | 358 (64.6) | 75 (60.5) | |
| Diffuse | 18 (48.6) | 196 (35.4) | 49 (39.5) | |
| Unknown | 0 | 42 | 2 | |
| Lymph node metastasis | | | | P < 0.005[a,b] |
| Positive | 14 (37.8) | 37 (6.4) | 50 (39.7) | |
| Negative | 23 (62.2) | 543 (93.6) | 76 (60.3) | |
| Unknown | 0 | 16 | | |
| Lymph vessel invasion | | | | P < 0.025[a]<br>P < 0.005[b] |
| Positive | 15 (40.5) | 37 (9.4) | 29 (43.9) | |
| Negative | 22 (59.5) | 357 (90.6) | 37 (56.1) | |
| Unknown | 0 | 202 | 60 | |
| Vascular invasion | | | | P < 0.005[a,b] |
| Positive | 4 (10.8) | 12 (3.1) | 13 (19.7) | |
| Negative | 33 (89.2) | 378 (96.9) | 53 (80.3) | |
| Unknown | 0 | 206 | 60 | |
| Survival | | | | |
| Alive | 34 | 489 | 91 | |
| Death | 5 | 107 | 35 | |
| Peritonitis carcinomatosis | 2 | 4 | 5 | |
| Liver metastasis | 2 | 3 | 7 | |
| Local recurrence | 0 | 0 | 1 | |
| Undefined recurrence | 1 | 11 | 10 | |
| Operative death | 0 | 4 | 1 | |
| Other disease | 0 | 85 | 11 | |

NS, not stastical; [a]EGCs mimicking AGC vs other EGCs; [b]mp gastric cancers vs other EGCs. Survivals of patients were investigated in June 1995.

**Figure 2** Subdivision of the submucosal cancers according to the extent of cancer invasion. (**A**) sm1, slight invasion limited to the upper submucosa; (**B**) sm2, moderate invasion into the middle of the submucosa; (**C**) sm3 deep invasion close to the muscular layer

**Figure 3** Typical histological findings of an sm3 tumour. Cancer cells invaded into the submucosal layer extensively

**Figure 4** Survival rates of patients with EGCs mimicking AGCs. The postoperative survival rate of patients with EGCs mimicking AGCs was intermediate between patients with other EGCs and mp gastric cancers

### The extent of submucosal invasion

Of the 37 EGCs mimicking AGCs and the other 596 EGCs, 30 and 320 cases, respectively, had cancer invasion to the submucosal layer of the stomach. The extent of submucosal invasion was examined in the 30 EGCs mimicking AGCs and in 100 solitary submucosal EGCs of another type. Submucosal invasion was classified into three subgroups according to the extent of invasion (Figure 2, Inove et al, 1991): (1) sm1, slight invasion limited to the upper submucosa; (2) sm2, moderate invasion into the middle of the submucosa and (3) sm3, deep submucosal invasion close to the muscular layer. Five (16.7%), eight (26.7%) and 18 (60%) lesions were classified as sm1, sm2 and sm3 respectively, in the EGCs mimicking AGCs. In contrast, 43 (43%), 34 (34%) and 24 (24%) lesions were classified as sm1, sm2 and sm3, respectively, in the other EGCs. Of the 14 node-positive EGCs mimicking AGCs, 11 cases (78.6%) were sm3. The typical histology of sm3 invasion is shown in Figure 3.

### Survival rate

The post-operative survival rate of patients with EGCs mimicking AGCs was intermediate between patients with other EGCs and patients with mp gastric cancers (Figure 4). The 5-year survival rates were 76%, 89% and 95% in patients with mp gastric cancer, those with EGCs mimicking AGCs and those with other EGCs respectively. The 10-year survival rates were 71%, 87%, and 92% in mp gastric cancer, EGCs mimicking AGCs, and the other EGC groups. There was a significant difference in the 5-year and 10-year survival rates between the other EGC patients and mp gastric cancer patients ($P < 0.05$), but no difference in those rates between patients with EGCs mimicking AGC and mp gastric cancer patients.

### DISCUSSION

The most predominant pathological feature of EGCs mimicking AGCs was a higher incidence of lymph node metastasis in comparison with the other types of EGCs; 37.8% for EGCs mimicking AGCs versus 6.4% for the other EGCs. Several factors have been reported to be correlated with lymph node metastasis in gastric cancer: tumour size, the depth of invasion, lymph vessel invasion and histological type (Hioki et al, 1990; Maehara et al, 1992). Our study revealed that, of these factors, a greater tumour size, massive sm invasion and frequent lymph vessel invasion were more prominent in EGCs mimicking AGCs than in the other EGCs. In our previous report, a multivariate analysis using a logistic regression adjustment showed that among these three factors, the last two were independent risk factors for lymph node metastasis in early gastric cancer (Kitamura et al, 1996*b*). In particular, massive sm invasion was strongly associated with lymph node metastasis in EGC. In the present study, massive sm invasion was particularly frequent when EGCs mimicking AGCs also had lymph node metastasis. This result is explainable by the observation that the submucosal layer of the stomach is rich in lymphatic capillaries (Inoue et al, 1991; Maehara et al, 1992; Sano et al, 1992).

We showed that 60% of EGCs simulating AGC had sm3 invasion, whereas 24% of the other EGCs had sm3 invasion. The rate of sm3 invasion in EGCs mimicking AGCs is apparently higher than that of the other EGCs; this has also been described in other reports (Miyamoto et al, 1987; Ikeguchi et al, 1989). We also showed that the rate of sm3 invasion accounted for 78.6% of EGCs mimicking AGCs when they were complicated by lymph node metastasis. The higher frequency of sm3 invasion in EGCs mimicking AGCs indicates that these tumours readily produce massive sm invasion, and as a result lead to more frequent lymph node metastasis.

The histological findings of the EGCs mimicking AGCs mentioned above were strongly connected with their macroscopic appearance; 16 of the 37 cancers had a macroscopic appearance of an unclassified type according to the Borrmann classification. The macroscopic appearance was characteristic of a greater tumour size, the presence of a deep ulcer and scirrhous changes in the gastric wall. We found that the cancer lesions with a deep ulcer and scirrhous changes were often accompanied by histological destruction of the normal gastric architecture. This histological destruction might allow the cancer cells to invade into the deeper zone of the gastric wall and to produce massive sm invasion. Thus, the macroscopic appearance described above is a useful predictor for massive sm invasion and lymph node metastasis in early gastric cancer.

Patients with EGCs mimicking AGCs showed an intermediate post-operative survival rate between patients with mp gastric cancer and those with other EGCs. Because of the limited number of patients with EGCs mimicking AGCs, it is currently difficult to calculate their post-operative prognosis precisely. However, we believe that the post-operative prognosis in patients with EGCs mimicking AGCs is similar to that in patients with mp gastric cancers, because the clinicopathological findings are very similar in the two groups. This congruence of clinicopathological findings would be reflected by the biological behaviour of the tumour in groups, and also by their post-operative survival rate. Above all, it is worth mentioning that the frequency of lymph node metastasis was similar in the two groups. Lymph node metastasis is the most reliable prognostic factor in gastric cancers when the depth of invasion is similar (Maruyama et al, 1987; Hioki et al, 1990; Maehara et al, 1992; Sano et al, 1992). The results of the clinicopathological findings indicate that EGCs mimicking AGCs should be treated in a similar fashion to mp gastric cancers. Extensive lymph node dissection should be performed on EGCs mimicking AGCs as well as on mp gastric cancers.

© Cancer Research Campaign 1997

Recent advances in diagnostic techniques allowed us to determine preoperative and intraoperative staging of gastric cancer (Tatsuta et al, 1982; Yasuda et al, 1986; Tio et al, 1989). In particular, endoscopic ultrasonography and intraoperative histological examination are useful for more objective determination of the depth of invasion for gastric cancer, which is strongly correlated with patients' survival time (Maruyama et al, 1989; Kitamura et al, 1996*c*). Preoperative and intraoperative assessment of the depth of invasion is imperative for determining the surgical procedure: limited or extensive lymph node dissection. In recent years, we have routinely used endoscopic ultrasonography to gauge the depth of invasion, and sometimes intraoperative frozen section. These examinations will become more important for determining the appropriate surgical procedure for early gastric cancer in future. However, these examinations may be rather disadvantageous for determining the appropriate surgical procedure for EGCs mimicking AGC, considering their clinicopathological features. These cancers should be treated as advanced gastric cancer if they were diagnosed with early gastric cancer by endoscopic ultrasonography and intraoperative histological examination.

In conclusion, the present study demonstrated that macroscopic appearance is a useful predictor of massive sm invasion and lymph node metastasis in EGC. As EGCs mimicking AGCs are similar to mp gastric cancers in their clinicopathological features, these cancers should be treated as mp gastric cancers.

### REFERENCES

Hioki K, Nakane Y and Yamamoto M (1990) Surgical strategy for early gastric cancer. *Br J Surg* **77**: 1330–1334

Ichiyoshi Y, Toda T, Minamisono Y, Nagasaki S, Yakeishi Y and Sugimachi K (1990) Recurrence in early gastric cancer. *Surgery* **107**: 489–495

Ikeguchi M, Yonekawa M, Ohta M, Sumi K, Makino M, Kimura O, Nishidoi H, Kaibara N and Koga S (1989) Role of the lamina muscularis mucosae on submucosal invasion of gastric cancer. *Jpn J Gastroenterol Surg* **22**: 2333–2337

Inoue K, Tobe T, Kan N, Nio Y, Sakai M, Takeuchi E and Sugiyama T (1991) Problems in the definition and treatment of early gastric cancer. *Br J Surg* **78**: 818–821

Japanese Research Society for Gastric Cancer (1981) The general rules for the gastric cancer study in surgery and pathology. *Jpn J Surg* **11**: 127–139

Kitamura K, Hagiwara A, Otsuji E, Shimotsuma M, Taniguchi A, Yamaguchi T, Sawai K and Takahashi T (1995*a*) Activated carbon-oriented gastrectomy for early gastric cancer. *Br J Surg* **82**: 647–649

Kitamura K, Yamaguchi T, Okamoto K, Taniguchi H, Hagiwara A, Sawai K and Takahashi T (1995*b*) Total gastrectomy for early gastric cancer. *J Surg Oncol* **60**: 83–88

Kitamura K, Yamaguchi T, Taniguchi H, Hagiwara A, Yamane T, Sawai K and Takahashi T (1996*a*) Clinicopathologic characteristics of gastric cancer in the elderly. *Br J Cancer* **73**: 798–802

Kitamura K, Yamaguchi T, Okamoto K, Nishida S and Takahashi T (1996*b*) Superficial spreading type of early gastric cancer. *Br J Cancer* **74**: 183

Kitamura K, Yamaguchi T, Taniguchi H, Hagiwara A, Sawai K and Takahashi T (1996*c*) Analysis of lymph node metastasis in early gastric cancer: Rationale of limited surgery. *J Surg Oncol* **64**: 42–47

Maehara Y, Orita H and Okuyama T (1992) Predictors of lymph node metastasis in early gastric cancer. *Br J Surg* **79**: 245–247

Maruyama K, Okabayashi K and Kinoshita Y (1987) Prognosis in gastric cancer in Japan and its limits of radicality. *World J Surg* **11**: 418–425

Maruyama K, Gunven P, Okabayashi K, Sasako M and Kinoshita T (1989) Lymph node metastases of gastric cancer. *Ann Surg* **210**: 596–602

Miyamoto Y, Oowada S, Tanahashi Y, Kawai T and Izuo M (1987) Clinicopathological study of early gastric cancers with submucosal invasion (in Japanese). *J Jpn Soc Clin Surg* **48**: 589–594

Moreaux J and Bougaran J (1993) Early gastric cancer – A 25 year surgical experience. *Ann Surg* **217**: 347–355

Mori M, Sugimachi K, Ohiwa T, Okamura T, Tamura S and Inokuchi K (1985) Early gastric carcinoma in Japanese patients under 30 years of age. *Br J Surg* **72**: 289–291

Noguchi Y, Ohta H, Takagi, Ike H, Takahasi T, Ohashi I, Kuno K, Kajitani T and Kato Y (1985) Synchronous multiple early gastric carcinoma: A study of 178 cases. *World J Surg* **11**: 127–139

Ohta H, Noguchi Y and Takagi K (1987) Early gastric carcinoma with special reference to macroscopic classification. *Cancer* **60**: 1099–1106

Sano T, Kobori O and Muto T (1992) Lymph node metastasis from early gastric cancer: endoscopic resection of tumour. *Br J Surg* **79**: 241–244

Tatsuta M, Okuda S and Tamura H (1982) Endoscopic diagnosis of early gastric cancer by the endoscopic congo red–methylene blue test. *Cancer* **50**: 2956–2960

Tio TL, Schouwink MH, Cikot RJLM (1989) Preoperative TNM classification of gastric carcinoma by endosonography in comparison with the pathological TNM system: a prospective study of 72 cases. *Hepato-Gastroenterology* **36**: 51–56

Yasuda K, Kiyota K, Mukai H and Nakajima T (1986) Endoscopic ultrasonography (EUS) in the diagnosis of upper digestive tract diseases – determination of the depth of cancer invasion. Gastroenterol (in Japanese) *Endoscopy* **28**: 253–263

© Cancer Research Campaign 1997